# Tracing the clinicodermoscopic and histopathological evolution of acquired reactive perforating collagenosis: a case report

**DOI:** 10.3389/fmed.2026.1812713

**Published:** 2026-06-16

**Authors:** Jue Wang, Lizhu Chen, Xinlong Chen, Nuoyan Li, Mingling Chen, Zhifei Wen

**Affiliations:** 1 Department of Dermatology, Hospital of Chengdu University of Traditional Chinese Medicine, Chengdu, Sichuan, China; 2 Chengdu University of Traditional Chinese Medicine, Chengdu, Sichuan, China

**Keywords:** acquired reactive perforating collagenosis, case report, dermoscopy, diabetes related dermatosis, histopathological

## Abstract

Acquired reactive perforating collagenosis (ARPC) is a rare perforating dermatosis that is frequently misdiagnosed, particularly before the development of overt transepidermal elimination. Although early lesions have been sporadically mentioned in previous reports, direct clinicopathological evidence supporting a recognizable pre-perforating stage remains limited. We report the case of a 64-year-old woman with a 3-year history of progressive, generalized pruritic papules and nodules, initially misdiagnosed as eczema and refractory to conventional treatments. The patient had long-standing type 2 diabetes mellitus and other metabolic comorbidities. Dermoscopy of early erythematous papules revealed subtle but reproducible features distinct from those of fully developed lesions. Notably, even in early lesions showing focal epidermal disruption, classic dermoscopic signs of perforation were absent. Targeted biopsy of these early lesions exhibited abnormal aggregation and disorganization of dermal collagen in the superficial dermis, closely apposed to the epidermis but without transepidermal elimination. In contrast, biopsies of mature umbilicated plaques revealed typical collagen extrusion with overlying keratotic plugs. Sequential Masson’s trichrome and elastic fiber staining further supported a stepwise evolution from superficial collagen remodeling to overt perforation. By correlating dermoscopic findings with sequential histopathological changes in the same patient, this case report provides direct evidence that ARPC progresses through a definable pre-perforating stage, in which epidermal disruption alone is insufficient to induce collagen elimination. Recognition of this early stage has important implications for timely diagnosis, appropriate biopsy selection, and avoidance of prolonged misdiagnosis.

## Introduction

Acquired reactive perforating collagenosis (ARPC) is an uncommon perforating dermatosis characterized by transepidermal elimination of altered dermal collagen. It belongs to the spectrum of perforating dermatoses, which also includes elastosis perforans serpiginosa, perforating folliculitis, and hyperkeratosis follicularis et parafollicularis (Kyrle disease). These disorders share the histopathological feature of epidermal perforation with transepidermal elimination of dermal material, although their clinical presentation, associated conditions, and the predominant eliminated components differ. ARPC is most frequently associated with systemic metabolic disorders, particularly diabetes mellitus and chronic renal disease, and typically presents as intensely pruritic, umbilicated papules or nodules with firmly adherent keratotic plugs (1–3). Owing to its rarity and the frequent reliance on overt transepidermal elimination as a diagnostic hallmark, ARPC is often underrecognized or misdiagnosed before fully developed perforation becomes evident.

The current understanding of ARPC is largely derived from the histopathological examination of fully developed lesions, in which transepidermal collagen extrusion represents the diagnostic hallmark (2, 4). However, this feature may be absent in biopsies taken from early or evolving lesions, resulting in diagnostic uncertainty. Although early lesions have been sporadically mentioned in previous reports, direct clinicopathological evidence supporting a recognizable pre-perforating stage remains limited (4, 5).

Dermoscopy is a useful non-invasive adjunct in the evaluation of perforating dermatoses and may facilitate biopsy-site selection. Previous reports have described relatively consistent dermoscopic features in established ARPC, including a central yellow-brown or hemorrhagic structureless area, a surrounding white rim or collarette, and a peripheral erythematous zone with dotted, linear, or hairpin vessels (6–9). In a recent clinicopathological study of acquired perforating dermatoses, dermoscopy and reflectance confocal microscopy were considered helpful for rapid diagnosis and early intervention (10). However, dermoscopic descriptions of early pre-perforating lesions remain scarce, and their histopathological correlates have not been well defined. In this study, we report a case of ARPC in which dermoscopic findings and sequential histopathological features of early lesions were directly correlated with those of fully developed lesions in the same patient, providing evidence for a recognizable pre-perforating stage.

## Case presentation

**Figure 1:**
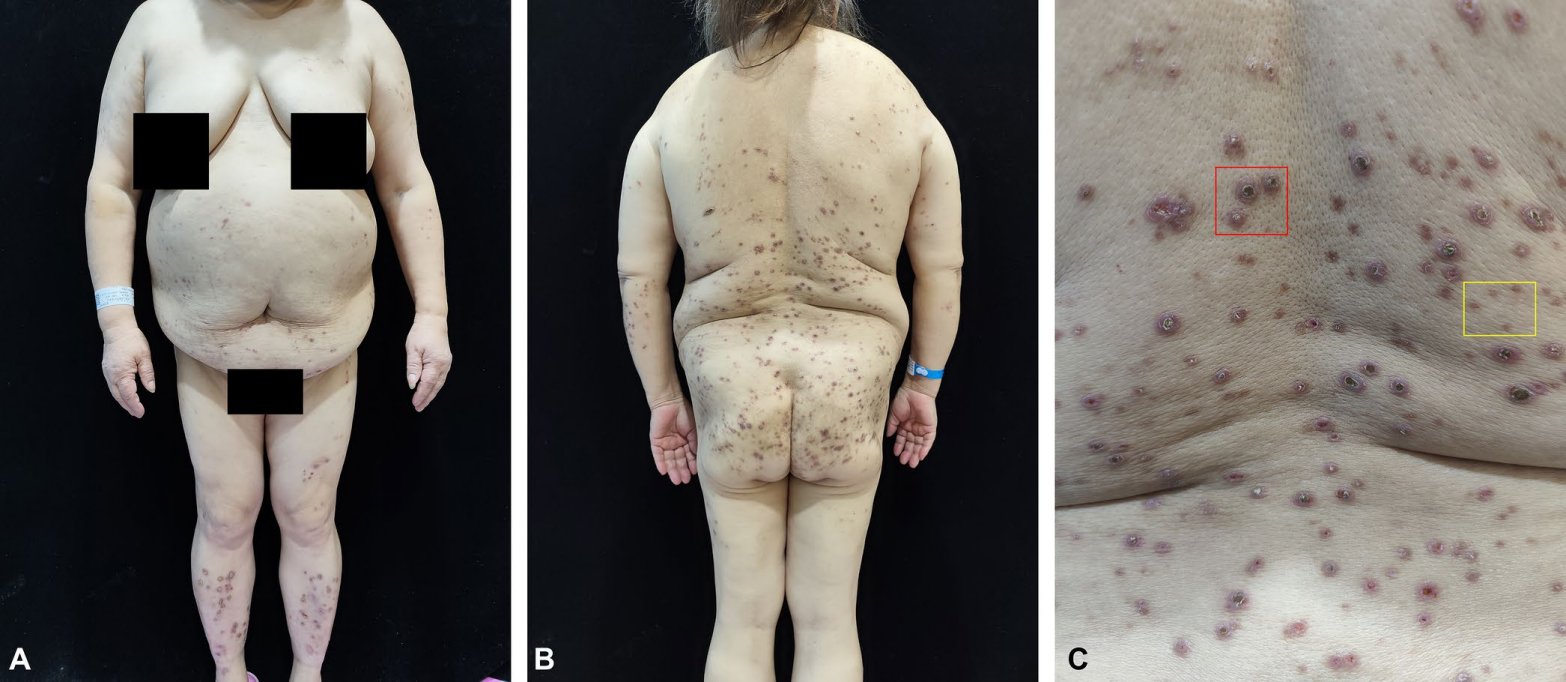
Clinical manifestations. **(A)** The anterior view shows multiple disseminated pruritic keratotic papules and nodules involving the trunk and extensor surfaces of the extremities. **(B)** The posterior view demonstrates widespread involvement of the back, buttocks, and lower extremities, with a predilection for extensor areas. **(C)** A close-up view of the lower back shows lesions at different stages of evolution. Lesions highlighted by the yellow box represent early erythematous papules without a well-formed central keratotic plug, whereas those highlighted by the red box indicate established lesions with central brownish keratotic plugs surrounded by erythema.

We report the case of a 64-year-old woman who presented with a 3-year history of progressive, generalized pruritic papules and nodules, with marked exacerbation over the preceding 2 months. The eruption was initially misdiagnosed as eczema, and both self-administered anti-inflammatory and antihistamine therapy, as well as subsequent inpatient treatment at a local hospital, failed to provide improvement. Her medical history was notable for type 2 diabetes mellitus for 20 years with suboptimal glycemic control, treated with sitagliptin, metformin sustained-release, and miglitol. She also had a more than 10-year history of hypertension, controlled with valsartan, hydrochlorothiazide, and coronary artery disease, managed with rosuvastatin and clopidogrel. No family history of similar skin disorders was observed. Physical examination revealed numerous disseminated hyperkeratotic papules, erythematous lesions, and bean- to peanut-sized nodules involving the trunk and extensor surfaces of the extremities, particularly the back and lower legs (Figures 1A–C). Many lesions exhibited raised borders with central umbilication containing a firmly adherent brown keratotic plug surrounded by erythema. The central keratotic material was hard and difficult to remove, and scattered atrophic scars were observed.

**Figure 2:**
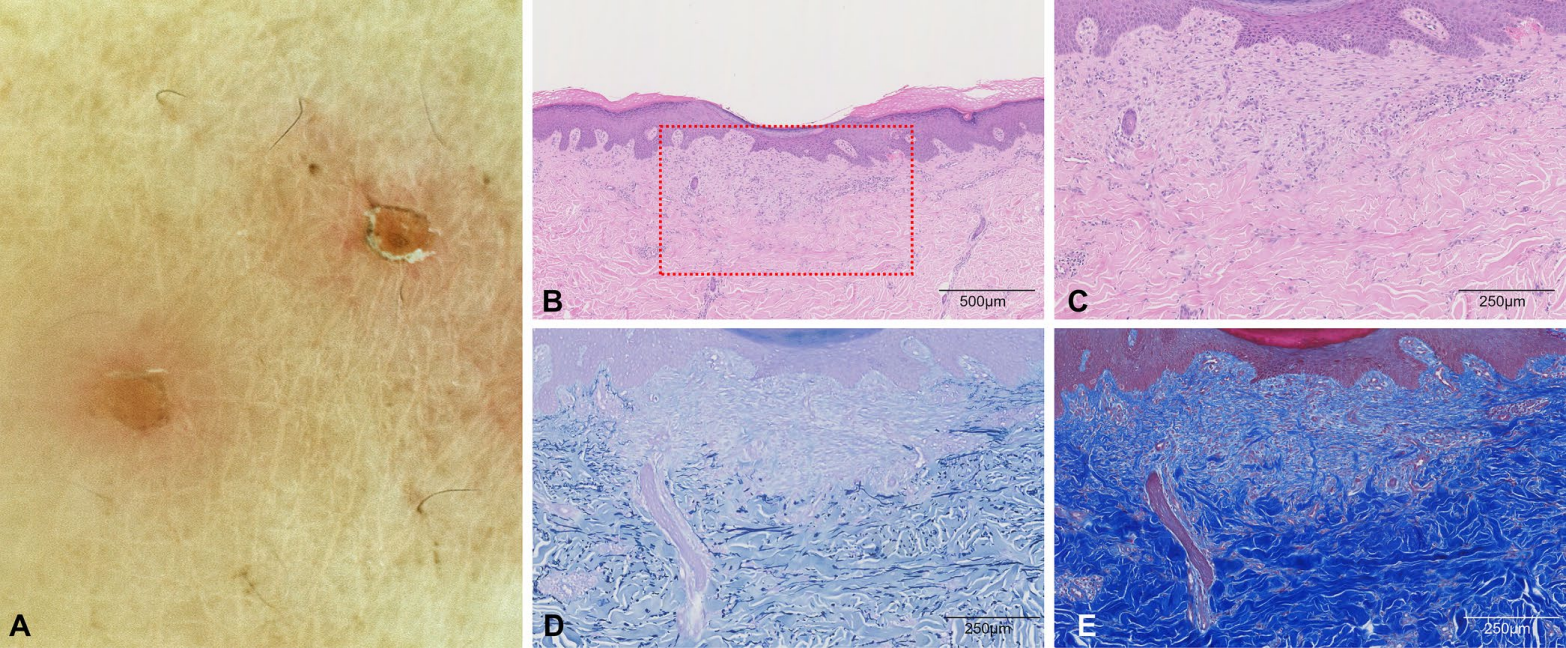
Clinicodermoscopic and histopathological features of early (pre-perforating) lesions. **(A)** A dermoscopic image of an early erythematous papule shows a faint central yellowish-white to light-brown structureless change with subtle surface attenuation on a pink background, without a well-formed keratotic plug or white collarette (×20). **(C)** Higher magnification of the boxed area in **(B)**. **(D)** Victoria blue elastic fiber staining of the same region. **(E)** Masson’s trichrome staining of the same region. Scale bars: 500 μm in **(B)**; 250 μm in **(C–E)**.

Dermoscopy was performed using a handheld dermoscope (DermLite, 3Gen, San Juan Capistrano, CA, USA) in polarized mode at ×20 magnification. Early erythematous papules showed a faint central yellowish-white to light-brown structureless area with subtle surface attenuation on a pink erythematous background, without a well-formed central keratotic plug, a hemorrhagic crust, a white collarette, or a complete three-zone concentric architecture (Figure 2A). No obvious radially arranged dotted or hairpin vessels were identified in these early lesions. Targeted biopsy of an early lesion revealed focal thinning and partial attenuation of the stratum corneum, with mild reactive changes at the dermal–epidermal junction, including subtle basal vacuolar alteration and sparse lymphocytic tagging. Beneath this, a band-like zone of structurally altered collagen fibers was observed in the superficial dermis. These collagen fibers appeared thickened, eosinophilic, and haphazardly arranged, with focal clustering immediately subjacent to the epidermis. A superficial perivascular and interstitial lymphohistiocytic infiltrate was present. Importantly, no cup-shaped epidermal invagination or transepidermal elimination was identified. The altered collagen fibers remained confined to the superficial dermis. Victoria blue staining showed no significant elastic fiber extrusion, and Masson’s trichrome staining confirmed superficial collagen fiber remodeling without vertical extrusion (Figures 2B–E).

**Figure 3:**
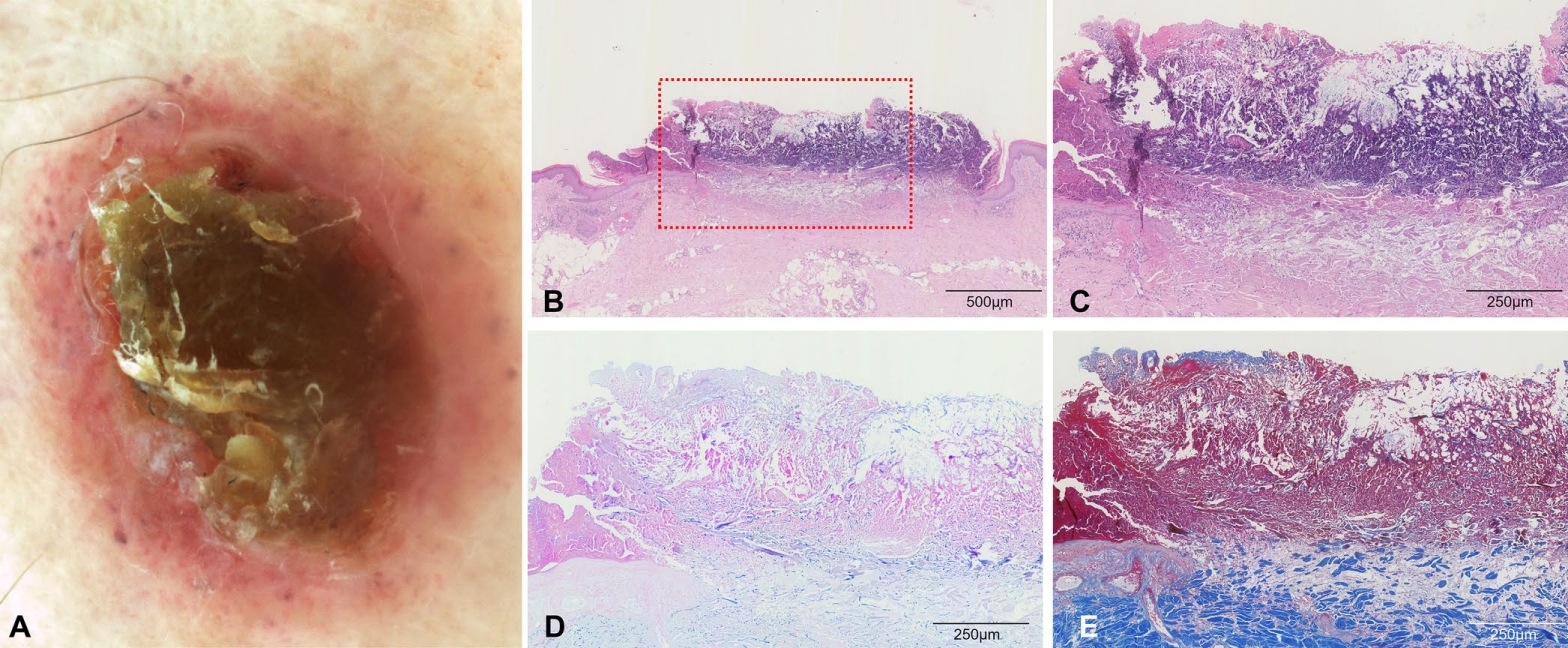
Clinicodermoscopic and histopathological features of fully developed perforating lesions. **(A)** A dermoscopic image of a mature lesion shows a central yellow–brown keratotic structureless area, a surrounding whitish collarette, and a peripheral erythematous halo (×20). **(B)** Low-power hematoxylin and eosin (H&E) staining of the corresponding lesion. **(C)** Higher magnification of the boxed area in **(B)**. **(D)** Victoria blue elastic fiber staining of the same region. **(E)** Masson’s trichrome staining of the same region. Scale bars: 500 μm in **(B)**; 250 μm in **(C–E)**.

In contrast, dermoscopy of mature lesions exhibited a prominent central yellow–brown to brown keratotic structureless area, surrounded by a whitish collarette or irregular white rim and a peripheral erythematous-pink halo; in some fields, small hemorrhagic spots and crusts contributed to an incomplete three-zone appearance (Figure 3A). Histopathological examination revealed a well-formed cup-shaped epidermal invagination filled with keratotic and necrotic debris. Eosinophilic altered collagen fibers extended vertically from the superficial dermis into the epidermal defect, consistent with established transepidermal elimination. These findings were confirmed by routine histology, as well as Victoria blue and Masson’s trichrome staining, which highlighted extrusion of disorganized collagen fibers through the epidermis, accompanied by secondary disruption of elastic fibers (Figures 3B–E).

Based on the combined clinical, dermoscopic, histopathological, and histochemical findings, a diagnosis of ARPC was established. The clinical background of long-standing diabetes mellitus, together with transepidermal elimination of altered collagen highlighted by Masson’s trichrome staining, supported this diagnosis. Elastosis perforans serpiginosa was considered unlikely because Victoria blue staining did not show primary elastic fiber elimination. Perforating folliculitis was also excluded because the perforating process was not folliculocentric, and Kyrle disease was considered less likely because the eliminated material was collagen rather than predominantly keratotic or basophilic debris.

## Discussion

The present case demonstrates a clear histopathological continuum in ARPC, linking early non-perforating lesions to fully developed perforating lesions within the same patient. The direct intra-individual comparison supports the concept that transepidermal elimination represents a later event in lesion evolution rather than the initiating pathological process.

A key diagnostic challenge in ARPC lies in distinguishing genuine early disease from the sampling artifact. One concern is whether the absence of transepidermal elimination simply reflects incomplete sectioning. However, in this case, multiple consecutive sections from the targeted early-lesion biopsy consistently showed superficial eosinophilic collagen remodeling immediately beneath a thinned but non-invaginated epidermis, without evidence of collagen extrusion. By contrast, biopsies from fully developed lesions in the same patient showed unequivocal vertical extrusion of altered collagen through a well-defined epidermal channel. This sequential intra-individual comparison strongly supports a true temporal progression of histopathological alterations rather than a technical limitation.

Our findings further suggest that the absence of transepidermal elimination does not equate to the absence of diagnostic value in early ARPC. In the early lesion, collagen bundles were abnormally positioned close to the dermal–epidermal junction and exhibited structural disorganization and eosinophilic alteration. Although such findings may be considered non-specific when viewed in isolation, the localized band-like zone of superficially aggregated altered collagen is not a typical feature of common eczematous dermatitis, in which dermal collagen architecture is usually preserved despite inflammatory changes. In the appropriate clinical setting, this pattern may therefore represent a disease-relevant pre-perforating alteration. Special stains further supported a stepwise pathological process, confirming collagen remodeling in the early lesion and overt transepidermal extrusion in the mature lesion, while arguing against a primary elastic perforating process.

Dermoscopy played a pivotal role in identifying lesions suitable for stage-specific biopsy and supports the concept that ARPC shows stage-related variation rather than a single uniform pattern. Previous reports of established ARPC have described relatively consistent mature-stage findings, including a central yellow–brown or hemorrhagic structureless area, a surrounding white rim or collarette, and a peripheral erythematous zone with vascular structures; in some lesions, hemorrhagic spots or crusts contribute to a concentric or three-zone appearance (6–9), 10. Elmas et al. (7) further suggested stage-related variation by describing five dermoscopic patterns, with patterns 1 and 2 more likely representing well-established lesions and others corresponding to excoriated, late, or healed lesions. In our case, the mature lesions were consistent with these published descriptions. Importantly, the early lesion did not lack dermoscopic features; rather, it showed a distinct early-stage pattern characterized by a faint central yellowish-white to light-brown structureless area with subtle surface attenuation on a pink erythematous background. Although this pattern lacked the fully developed keratotic or hemorrhagic center, well-formed white collarette, and complete three-zone architecture seen in mature lesions, it still represented a recognizable dermoscopic clue. Histopathologically, this early dermoscopic pattern corresponded to superficial altered collagen aggregation beneath the epidermis without transepidermal elimination. Therefore, our case not only supports stage-related variation in ARPC but also expands the existing literature by suggesting that early lesions may already display identifiable dermoscopic features before overt perforation develops. We intentionally phrase this as a plausible early clue rather than a definitive diagnostic criterion, as broader validation is still needed.

Several limitations warrant consideration. This report describes a single patient, and validation in larger cohorts is necessary. Moreover, while metabolic factors and scratching are frequently implicated in ARPC, this study does not address pathogenetic mechanisms but rather documents morphological evolution across disease stages.

## Conclusion

This case provides direct evidence that ARPC progresses through a definable pre-perforating stage characterized by altered dermal collagen fibers beneath the epidermis without transepidermal elimination. Correlation of dermoscopy with sequential histopathology enables recognition of this early stage, highlighting that reliance on transepidermal elimination alone may delay diagnosis, while early identification of superficial collagen alteration can guide timely biopsy and appropriate management, thereby avoiding prolonged misdiagnosis. Future studies are warranted to determine whether these early features can be incorporated into practical diagnostic criteria for ARPC.

## Data Availability

The original contributions presented in the study are included in the article/supplementary material, further inquiries can be directed to the corresponding author.
